# Understanding Behavioural Development of Calves in Natural Settings to Inform Calf Management

**DOI:** 10.3390/ani11082446

**Published:** 2021-08-20

**Authors:** Laura Whalin, Daniel M. Weary, Marina A. G. von Keyserlingk

**Affiliations:** Animal Welfare Program, Faculty of Land and Food Systems, University of British Columbia, 2357 Main Mall, Vancouver, BC V6T 1Z4, Canada; laura.whalin@alumni.ubc.ca (L.W.); danweary@mail.ubc.ca (D.M.W.)

**Keywords:** dairy calves, animal welfare, calf housing, feeding behaviour, social behaviour

## Abstract

**Simple Summary:**

Animal welfare research is intended to address societal concerns regarding animal care, including natural living concerns. We reviewed the literature on calf behaviour when reared in more naturalistic outdoor systems with their mothers and herd. Understanding calf behaviour in more natural settings may help inform changes in calf management and housing that promote behaviours important to calf welfare.

**Abstract:**

One important type of animal welfare concern is “natural living” (i.e., that animals are able to express natural behaviours that are important to them, and to engage with aspects of the natural world that they find important). The aims of this narrative review were to describe the behavioural development of calves (*Bos taurus*) in natural settings and use this to identify characteristics of natural systems that may be important to consider relative to this natural living conception of animal welfare. At birth, calves are licked by their mothers and soon stand to suckle for colostrum, and during the milk-feeding period, calves spend much of their time lying down. In natural systems, calves perform a variety of social behaviours with herd-mates, and slowly transition from their mother’s milk to eating solid food, by gradually increasing time spent grazing and ruminating. In contrast, on most commercial dairy systems, dairy calves are removed from their mothers at birth, housed individually, fed restricted amounts of milk and weaned abruptly at a young age. The results of this review suggest that accommodating key natural behaviours, for example through the use of teat feeding of milk, social housing, and gradual weaning, can help address welfare concerns.

## 1. Introduction

One way of interpreting animal welfare is Fraser and colleagues’ [1] model of three overlapping concerns: an animal’s biological functioning, affective states, and natural living. The latter is described as allowing an animal to use his/her adaptations when needed, and develop normally. Including opportunities for natural behaviour in animal systems may allow each animal to meet his or her needs, engage in positive experiences, and develop behaviourally [2]. Citizens frequently raise concerns regarding naturalness [3,4], often calling for reasonably natural living environments that provide animals with sufficient space to engage in important behaviours such as grazing [5]. Natural living concerns are acknowledged as important, but challenges remain in how to promote these in modern systems because “natural” behaviours are variable, some may be detrimental to welfare, and because the concept is difficult to define [2].

Little progress has been made in understanding and incorporating aspects of natural living into modern management systems for dairy calves. Recently, Cantor et al. [6] compared the maternal, social, and nutritional environment of calves in conventional systems to those in wild environments, but these authors did not describe the behavioural development of calves in naturalistic settings. To our knowledge, no published review has evaluated the literature on the behavioural development of the calf in naturalistic conditions. Understanding calf behaviour in natural conditions may inform farm management changes intended to promote welfare. The aims of this review were to critically review the scientific literature on behaviours key to the normal development of calves in natural settings, and to identify general characteristics that could be incorporated into dairy calf (*Bos taurus*) management to improve welfare. Within each section, we first describe calf behaviours in a naturalistic setting (i.e., extensively living on rangeland or pasture) from birth to weaning, and contrast these with what is commonly observed in current management practices. We conclude by suggesting ways that calf management practices may be modified to facilitate key natural behaviours. We have focused our examples of current dairy practices primarily on dairy systems in Canada and the US, given that this is where we have most experience. However, on occasion, we also use examples from other parts of the world, and different species. Cattle were domesticated approximately 6000–8000 years ago [7] providing ample opportunity for changes in behaviour. Different species and breeds have adult behaviour characteristics that may differ (e.g., antipredator response, estrus behaviours, excitability, heat stress); thus, human–animal relationships and optimal climates may also differ [8]. We acknowledge the durations of behaviours different calf species perform may differ, but to our knowledge, no study to date has described differences in calf behaviour by breed or subspecies. Indeed, several studies have included descriptions of calf behaviour in pasture settings with a herd to understand behavioural patterns frequently discussed in the indoor housed dairy calf literature (e.g., play [9,10], suckling [11], social behaviours [12]). This literature review describes general shifts in behaviour as calves age, with evidence from general changes in wild ungulate behaviour (e.g., young bison, water buffalo, and reindeer), and supports older descriptive studies with more recent experimental work with calves reared in indoor settings. All general behaviours described in this review have been described in calves reared intensively (i.e., sucking, resting, play, social behaviours, grazing or eating hay, and ruminating), and this review highlights the importance of environment in facilitating these behaviours.

## 2. Calf Behavioural Development

### 2.1. Birth

#### 2.1.1. Cow–Calf Contact

Some, but not all, cows distance themselves from other adults in the herd before calving in both natural and farm settings [13,14,15]. Despite this variability, isolation-seeking behaviour of cows has been used to classify calves as “hiders” in the hider–follower paradigm [6,16,17]. For cows that do hide, both cow and calf normally rejoin the herd within a few days, suggesting that this isolation phase is short and calves are better classified as “followers” [18,19], with some arguing that calves can be both “hiders” and “followers” [20]. Immediately following birth, calves engage in head shaking, snuffling, and sneezing to remove amniotic fluid from their air passageways [21]. The mother vigorously licks the calf to remove amniotic fluid [22], establish the cow–calf bond (reviewed by von Keyserlingk and Weary [17]), and stimulate urination and defecation [23]. Licking soon after birth is seen in wild ungulates, such as free-ranging reindeer (*Rangifer tarandus* L.) and wild plains bison (*Bison bison*) [24,25]. When permitted, cows in loose housing barns begin licking their calf on average 5 [26] to 7 min after parturition [14]. Young ungulates typically spend most of the first hour after parturition being licked; for example, one study showed that Friesian calves born in individual pens were licked 30–50% of the time during their first hour of life [16], and Danish Holstein calves born indoors have recently been reported to be licked the first 40 min after birth [27].

In contrast, most dairy production systems remove newborn calves from their mothers within 12–24 h of birth (e.g., Brazil [28], United States [29], Australia [30]). There has been increasing interest in the effects of housing design at calving [15,27,31] and the effects of cow–calf contact in dairy systems [32,33,34], though research focusing on calf behaviour and health in these systems is still limited (reviewed by Johnsen et al. [35]).

#### 2.1.2. Standing

Wild ungulates are precocial; many young ungulates are able to stand and walk within an hour after birth so that they can suckle soon after birth [18]. Wild plains bison calves in the U.S. were observed to attempt standing approximately 3 min after birth, and on average, required only 11 min to successfully stand [25]. Water buffalo calves (*Bubalus bubalis*) in Australia attempted to stand 10 min after birth, and stood within 50 min of birth [36], and reindeer calves in Sweden also stood soon after birth (attempted to stand 5–29 min after birth, and stood 10–40 min after birth [24]). Dairy calves born indoors seem to behave similarly to their wild counterparts in this respect. Calves attempt to stand on average 17 min after birth, and successfully stand after an average of 37 [14] to 58 min [21]. Calves from dystocia births may have reduced vitality, possibly causing a reduced motivation to stand (reviewed by Murray and Leslie [37]). Calves with primiparous mothers have been reported to take longer [21] and spend less time standing during their first 6 h of life than calves from multiparous cows [16]. Though it has been reported that standing time during the first 6 h of life is not correlated with the amount of licking the calf receives from the mother, it is recognized that some primiparous cows in individual indoor pens never lick their calves, and some primiparous cows show fear towards their calf, which may affect standing time [16].

#### 2.1.3. Suckling

The ability to absorb the immunoglobulins from colostrum is highest immediately after birth, and this ability may decrease even 6 h after birth [38]. Thus, the need to stand soon after birth and suckle quickly is beneficial for the calf, and is seen across ungulate species. Wild ungulates, on average, take less than 90 min to begin suckling (reindeer: on average 82 min [24]; wild plains bison: on average 32 min [25]). In dairy cattle born indoors, the newborn will often nuzzle different parts of the cow’s body before finally locating the teat [14,16]. In cow–calf pairs housed separately from the group indoors, beef calves suckled their mothers on average 81 min after birth [21]. In contrast, although 9 of the 11 dairy calves observed in a loose-housing barn contacted their mother’s udder, only 4 (36%) of the calves successfully suckled within 3 h of birth [14]. Age of the cow may play a role in suckling success; 61% (17 out of 28) and 89% (25 out of 28) of Friesian calves from first lactation mothers suckled within 2 and 6 h of birth, unlike calves from multiparous mothers, where only 15% (8 out of 54) and 54% (29 out of 54) suckled within 2 h and 6 h, respectively [39]. These differences may be because the older Friesian cows had pendulous udders that limited the calf’s ability to suckle [39]. One study classified udder shape as either “good” (optimal for suckling) or “poor” and found that calves spent 17 min teat seeking before suckling from a “good” udder compared to 40 min for calves trying to suckle from a “poor” udder [21]. As research in cow–calf systems continues to burgeon, different methods for ensuring adequate suckling and colostrum intake should be described.

Interestingly, in group calving facilities (covered yard), as many as 54% of newborn dairy calves were reported to have been licked by a cow that was not their mother, and one-third of newborn calves suckled from these “alien” cows [22]. Edwards [22] suggests that calving in group pens may distract the cow and calf from suckling. Recent work has offered dairy cows housed indoors opportunities to self-isolate near parturition [40,41,42]. Most of these studies focused on the cow’s behaviour and preferences, but there is evidence that when cows and their calves have access to secluded spaces, calves do not suckle alien cows [41], and cows and calves interact less with other animals in the group [15]. However, there is individual variation in isolation seeking at parturition in dairy cows as well as cows in extensive systems [14,15,19]; more work is needed to elucidate the importance of the physical and social environment at birth and how this may influence suckling.

### 2.2. Pre-Weaning

#### 2.2.1. Resting

Regardless of system, young calves spend the majority of their time resting [43,44], and lying time declines as calves age [13,45,46]. In a study where zebu cows and calves were managed with 11 h of pasture access/day (d) and were otherwise confined to a covered yard, calves spent approximately 5 h/day lying during their first month of age, but this declined to less than 3 h/d lying and mimicked the resting schedules of their mothers by the time the calves were 4 months (mo) of age [45]. Kerr and Wood-Gush [46] observed beef calves reared on pasture with their mothers from 08:00 to 18:00 h and reported that at birth, the calves lay down about 65% of the time, and this reduced to 30% at 8 weeks (wk) of age. Interestingly, data collected during the night indicated that zebu calves spent most of the night resting (approximately 10 h/night during the first 2 mo of life [45]). Again, resting declined as calves aged; the authors noted a 60–90 min decline in resting during the night and an associated increase in time spent eating silage [45].

#### 2.2.2. Activity

When reared extensively with maternal contact, a calf’s activity may be directed by his or her mother. For example, when housed with daytime pasture access, 4 wk or older Zebu calves had similar walking durations as their mothers (both cows and calves spend approximately 2 h/d walking during the calf’s first 6 mo of age [45]). In contrast, beef calves younger than 4 mo of age walked less than their mothers [43]. However, these studies may not be directly comparable as Hutchison et al. [45] and Dwyer [43] studied different breeds of cattle with different space allowances (slopes and gullies with different vegetation of an unmeasured area provided during day and yard at night [45]; 1500 acres of variable topography and vegetation 24/7 [43]).

Calves also engage in play behaviours which increase in frequency over the first 2 wk of age [47]. Wild Maremma calves (*Bos primigenius taurus*) and Buran calves (*Bos indicus*) in extensive systems play by head butting, mounting, jumping, and running; and often, these behaviours are integrated into extended play bouts [13,24,48]. Free-ranging reindeer calves also play by chasing other calves, jumping, and butting objects around them [24]. The amount of space provided may affect these behaviours. One study found that 5 wk old calves raised in indoor group pens with more space (4 or 3 m^2^/calf) performed more play behaviour than calves in a smaller group pen (2.2 or 1.5 m^2^/calf [10]). Providing access to a large space may be important for calves to express their natural levels of walking and playing.

There is some evidence indicating that calves play in a diurnal pattern. Beef calves on pasture form small groups of 5–6 and play by running and kicking during the evening [43]. Similarly, zebu calves reared together with their mothers with daytime pasture access showed a peak in play between 16:30 and 18:30 h [49], and reindeer calves usually played between 18:00 and 21:00 h [24]. Play behaviour may also be influenced by hunger; for instance, zebu calves in a restrictive suckling system with pasture access [50] and semi-wild Maremma calves [13] played primarily after eating. Experimentally, indoor housed dairy calves fed a higher milk allowance (9 or 12 litres (L)/d) played more than calves fed a lower milk allowance (5 or 6 L/d; [51,52]). Thus, space provided, time of day, and hunger all contribute to play behaviour.

#### 2.2.3. Social Behaviours—With Adults

The first important social partner for the calf is his or her mother. In the first 2 wk of life, dairy calves housed indoors will increase their social behaviours towards their mothers, a shift that may indicate that calves become the initiator of contact [47]. Social cohesion between calf and mother may be initially driven by the calf’s need for milk, but there may also be calf factors. For instance, free-ranging female beef calves spent more time near their mothers than bull calves [20]. Age also influences the time cows and calves spend together. Both Maremma [13] and beef [46] calves on pasture appear to interact less with their mothers as the calves age, perhaps because they are becoming more independent.

In addition to being able to suckle, there may be other benefits to the cow–calf relationship, given that one study reported that dairy cows and calves indoors spent as much as 30% of their time together not suckling [33]. A calf mimics his or her mother’s daily activities [43,45] perhaps to learn. For example, reindeer calves grazed close to their mother to learn which plants to consume [24]. In sheep, social models play a role in dietary selection; for instance, lambs avoided the same plants that their mothers avoided [53,54]. Experimental work in cattle has found that naïve dairy heifers take less time to start grazing [55] and ruminate more on pasture when kept with an experienced animal [56]. Social learning, especially through modelling or learning by observing another individual, allows young animals to avoid dangerous mistakes (reviewed by Bandura [57]). Calves in close contact with the mother are also afforded protection; Dwyer [43] described beef calves on a prairie as staying close to their mothers during rain and unusual events.

Allogrooming appears to maintain the bond between the cow and calf. When dairy cows and calves were housed together for 12 h/d indoors, pairs spent approximately 10% of their time allogrooming [33]. Cows are able to lick and groom areas that the calf is unable to reach, such as the head, ears and neck [13,48], which may improve the coat hygiene of calves [58]. Calves reared in absence of their mother indoors showed more self-licking than calves raised with their mothers on pasture (approximately 6 vs. 3 instances/h [46]). Calves living with other calves also perform allogrooming but this behaviour is infrequent compared to cow–calf allogrooming (77 times over 164 days [48]; or 3–4 min per 12 h [59]). Although calves are frequently the recipients of grooming from their mothers, the value of this behaviour is not well understood. Some insights have been gained by investigating the use of brushes by young calves. When permitted, group housed calves indoors used brushes approximately 21 min/12 h [59] and paired calves did so nearly 30 min/20 h [60]. Interestingly, calves focused brush use on their head and neck [60], much like where the mother focused her attention when cows and calves were raised together [13,48]. One study that provided individually housed calves in hutches with multiple items (brush, artificial teats, pipe with molasses, and a rubber chain) found that calves used the brushes more than the other items, suggesting that the brush has particular value [61]. A brush may help stimulate grooming behaviour in dairy systems where calves are separated from their mothers, but more work is needed to understand the calf’s need for social grooming, and whether access to a brush provides a meaningful replacement for maternal grooming.

Calves also interact with other adults in the herd when provided the opportunity. Water buffalo present an interesting case. One study reported that most water buffalo within the herd (70%) came and sniffed new calves within 10 min of birth and this social cohesion continued throughout the calves’ development [36]. For example, on occasion, water buffalo herds formed groups of 4–13 calves under the supervision of 1–2 adult animals (adult cow, heifer, or young bull) while the other herd members left to graze [36]. Similarly, young beef calves on hill pasture have been reported to form groups of calves lying within 20 m of each other, which the authors labelled as crèches, though the authors were unable to determine if an adult animal guarded the calves [62]. Recently, researchers were able to systematically quantify *Bos indicus* calf groupings on pasture during their first month of age (≥3 individuals within 10 m [63]). Orihuela et al. [63] observed that most groups were 1 to 3 cows with 2 to 32 calves (65.4%; 93/142), with no groups consisting only of cows, and only 3.5% of the groups comprised of only calves (5/142). The proportion of calves in the group was usually higher than the proportion of cows (75.1%; 103/142) providing further indication of communal supervision for calves [63]. Adult water buffalos are highly social with evidence that orphan water buffalo calves are adopted by others in the herd [36]. There has been little work describing the complex social behaviour development of beef and dairy calves housed with adult animals other than their mothers. Of the limited research exploring calf and adult interactions, most pertain to suckling. For example, Simmental heifers within 8 h of calving have been reported to lick and sometimes nurse alien calves when housed in covered group pens [26]. Most calves raised with cow contact in indoor groups will suckle from another cow [33,64,65], a behaviour that is also seen in reindeer where cows were observed nursing other calves [24].

#### 2.2.4. Social Behaviours—With Calves

There is variation in when calves begin spending time with other calves. Free-ranging reindeer calves begin resting with other calves at 1 mo of age [24]. In contrast, beef calves on pasture spend increasing amounts of time with other calves from birth to 2 mo of age [46] and Maremma calves spend nearly an hour each day with other calves by 10 d of age [13]. Calves spend a considerable amount of time in the presence of other calves when they are 10–40 d of age (78–85 min/11 h [13]) but this appears to then wane [65], perhaps because play frequency declines after 6 wk of age (e.g., reindeer calves [24]). Group-housed dairy calves indoors also have been reported to play less as they age [10]. Only one study has attempted to describe social behaviours in calves (Buran) in a natural environment; Reinhardt et al. [48] followed calves from ages 4–6 mo to 9–11 mo and found that calves performed flehmen, pushing, butting, and licking, but rarely engaged in threatening or aggressive behaviours. Buran calves, however, spent the majority of their time grazing and resting and did so with preferred partners [48]. Experimental work has also investigated social bonding of calves using standardized tests [66], and some work has explored social proximity as a metric to explain individual differences [67], but the behaviour of calves in social settings has received little attention, especially social behaviour development, and the individual sociability of the pre-weaned calf.

Calves may have specific roles while playing with other calves. Young, semi-wild Maremma calves run and jump more than older calves, perhaps using play as physical training [13]. In semi-wild Maremma calves, males initiated play more often than females (58 vs. 42% of bouts initiated [13]), and both male muskox calves (*Ovibos moschatus*) and Buran calves performed more mounting than female calves [48,68]. Buran calves on pasture appeared to have stable roles in a play bout, where one calf consistently butted, and the other calf received the butting [48]. The role of play in calf development has not been well studied, but play may be used to build social skills to thrive in herd settings. For example, as Buran calves aged, regardless of sex, the frequency of butting increased but mounting and head-butting behaviours decreased, suggesting that butting may transform into an agonistic behaviour [48]. In muskox calves housed in an outdoor paddock, head butting has been described as a form of agonistic behaviour, allowing the animals to establish and maintain dominance [68]. In natural systems, calves interact with other calves while playing, grazing, and resting.

#### 2.2.5. Social Housing for Dairy Calves

Unlike in natural systems where calves can engage with herd mates of varying ages, dairy calves are typically housed in individual pens until weaning (70% of calves in Southern Brazil [28]; 70% of pre-weaned heifer calves in the US [29]; 63% of farms in Canada rear heifer calves individually [69]). Farms using automatic milk feeding systems often house calves in groups [70], as do farms using more extensive rearing systems (e.g., Australia where only ~14% of calves are housed individually [30]). Given the complex social interactions calves have with conspecifics (e.g., allogrooming, play, and social learning), it is not surprising that, when compared to individually housed calves, pair or group housed calves show improved cognitive development [71,72], perform more play behaviours [73], and are less reactive to novelty [74,75,76]. More work is needed to understand the importance of adults (both related and unrelated) in social learning, allogrooming, and protection for the calf, and how to apply mixed-age groups in dairy systems.

#### 2.2.6. Suckling—Natural Suckling Behaviour

**Figure 1:**
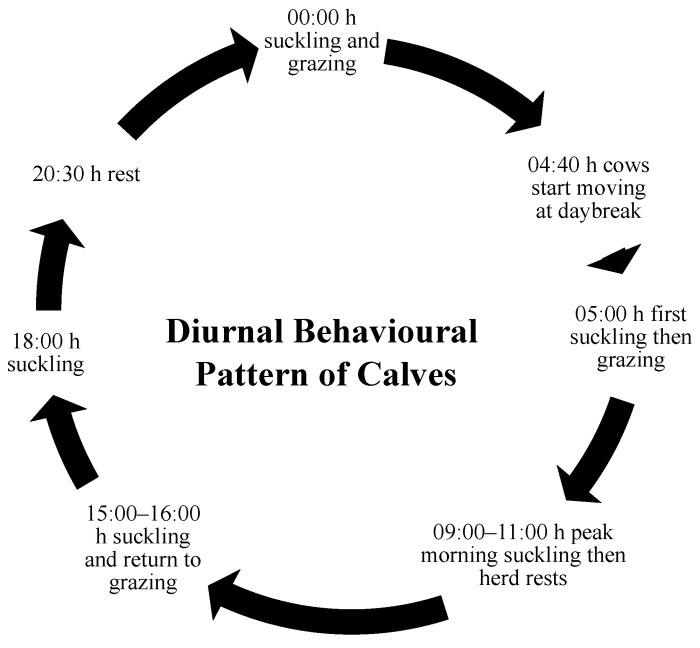
The diurnal behaviour pattern of calves raised with maternal contact on pasture in herds [13,43,45,49].

Cow–calf contact systems in natural settings (i.e., zebu, beef, Maremma on pasture or rangeland) appear to follow a consistent suckling pattern that occurs during behavioural transitions. Across several studies, calves were observed to suckle their mothers at dawn, mid-morning, mid-afternoon, evening, and again at midnight (Figure 1). More recent work found that suckling peaked at sunrise, just after noon (13:00–14:00 h), afternoon (15:00–16:00 h) and at night (22:00–23:00 h); however, the beef cattle in this study were housed in a barn likely limiting their activity [64]. At dawn, cows begin moving, and within 35 min, calves suckle for the first time that day [45,49,77]. Calves remain close to their mothers, grazing before suckling again mid-morning [43]. At this point, the herd often moves to find water and a resting place where they lie down for most of the early afternoon [13,43,78]. As the animals transition back to grazing mid-afternoon, calves will again suckle [13,43,49]. Calves usually engage in their final suckling bout when the herd transitions to resting at dusk [43,45,77]. There are a few studies that indicate that this pattern changes as calves age, with the introduction of grazing and a suckling event near midnight [43,77].

Calves raised with their mother (indoors and in pasture systems) will typically suckle in 8–11 min bouts [43,49,64], although suckling duration can increase with age [79]. When suckling from the mother, the calf will suckle one teat for a few seconds, then switch teats, with this sequence repeated until the bout ends (usually accompanied by a butting of each teat [43]). At 1 mo of age, calves engage in approximately 9–10 suckling bouts/d, and bout frequency decreases as the calf ages (at 4 mo, calves suckle 8 bouts/d, and by 6 mo, calves suckle 5–6 bouts/d; [45,79]). Several studies have reported that calves engage in fewer suckling bouts as they age [13,36,49]. Similarly, experimental work in dairy calves fed ad libitum milk from an artificial teat found that calves spent 13 min sucking/meal [80], visited the milk feeder approximately 9 times/d during their first 3 wk of life, and decreased their visits to the feeder with age [81]. During the first 2 wk of life, group-housed dairy calves raised indoors typically increase milk consumption and plateau at 15 L/d [81], and calves can consume more than 5 L in a meal [82].

#### 2.2.7. Suckling—Milk Feeding Management for Dairy Calves

Unlike in cow–calf contact systems where calves have free access to the cow, the majority of dairy calves are provided a restrictive milk allowance of approximately 4 L/d (southern Brazil: 4 L/d [28]; US: 57.7% of farms feed 3.78 L/d [29]; Australia: 4 L/d until 14 d, then 6 L/d until 79 d [30]). When fed restricted milk allowances, there is evidence that calves are hungry [83]. For example, when fed 4 L of milk/d, calves visited the milk feeder on average 24 times/d, more than 2.5 times the rate of calves fed ad libitum [81]. Similarly, when calves and cows are raised together, calves attempt to suckle more frequently when the cow has lower milk production [45]. Thus, suckling attempts seem to reflect calf hunger.

Additionally, approximately 40% of farms in Southern Brazil [28] and 72% of pre-weaned dairy heifer calves in the US [29] are bucket-fed milk, a practice that does not support their natural suckling behaviour. Calves provided milk from a teat spend approximately 45 min/d sucking (44 min/d [84]; 47 min/d [80]), while bucket-fed calves spend only about 18 min/d drinking milk [84]. Additionally, calves fed from a teat will butt the feeding apparatus [85], a behaviour frequently observed in calves suckling their mothers [43].

Cross-sucking (i.e., sucking on other calves), and sucking of inanimate objects, is often observed when calves are bucket-fed milk (reviewed by de Passillé [86]) or hungry [87], and is rarely described in natural settings when calves are provided sufficient milk from their mother. Fröberg and Lidfors [65] found that dairy calves raised with their mothers never performed cross-sucking, while 11 of the 18 calves fed a high milk allowance (9 L/d) from an automatic feeder performed this behaviour. The automatic feeder dispensed the milk in small portions [65], so calves may have been motivated to continue drinking even after receiving their milk allotment [88]. It is also possible that 9 L/d was not enough to satisfy the calves’ hunger given that calves can drink approximately 15 L/d when given the chance [80,81]. Interestingly, while dairy calves raised with their mothers still spend time licking other calves and objects, they appear to spend less time sucking on other calves and objects compared to calves fed from artificial teats [89]. Once again, the mother may play an important role in the behavioural development of the young calf. Calves fed milk via an artificial teat also spend less time cross-sucking than calves fed via a bucket [85]. Calves are highly motivated to suck, and bucket feeding does not allow them to perform this behaviour. For example, when calves had a dummy teat plus ad libitum milk via a bucket or a teat, teat-fed calves used the dummy teat 1 min/d, but calves with a bucket spent 13 min/d using the dummy teat [84]. Short of providing calves access to their mothers, providing ad libitum milk via a teat appears to elicit the most natural milk sucking behaviours.

#### 2.2.8. Grazing

When raised in natural conditions, calves have access to a complex physical environment with features to navigate such as space to explore, different forages, varying topography and soil types, changing climatic conditions, insects, and shade sources [24,25,43]. Of these features, interacting with grass is the most studied. Reindeer calves have been reported to engage in grazing or attempting to graze in their first day of life [24], and water buffalo calves nibbled grass by 8 d of age [36]. Grazing time increases with age; at 1 mo of age, calves spend approximately 2 h/d grazing (beef [77], zebu [45]), grazing then increases from 4–6 h/d to almost 9 h/d when calves are 2 and 4 mo old, respectively [43,45]. A more recent study found that beef calves 5–15 mo of age spent 5–7 h/12 h grazing [90]. Calves likely increase their grazing time as they age to meet their growing nutritional requirements as has been studied experimentally when calves transition from milk to solid feed (reviewed by Khan et al. [91]).

Much like suckling behaviour, grazing in calves also appears to also follow a diurnal pattern. Beef calves without maternal contact grazed at dawn, just before and just after noon, and again at dusk [77]. More recent work with maternally raised Nellore (*Bos indicus*) calves found that grazing was concentrated in the morning and early afternoon [78]. Older animals were reported to graze in a diurnal pattern with peaks in the morning and late afternoon to evening [90,92]. Though beef calves continued to graze in light rain [77], little is known concerning the effects of temperature, humidity, and wind on calf grazing behaviour, and use of the outdoors. Research has demonstrated how adult cattle respond to different conditions. Rangeland managed cattle will seek shelter in decreasing temperatures and rainy days [93], and when given the choice, dairy cows prefer the outdoors (on pasture [94], or a deep-bedded open pack [95]) at night during the summer.

Grazing behaviour can be affected by the provision of additional feed stuffs. Research in adult beef cattle found that adults preferred pasture to feedlots, regardless of the herbage mass available on pasture [96]; similarly, dairy cows showed a preference for pasture over being inside a freestall barn both when concentrated feed was provided outside and when it was not [97]. Not surprisingly, when calves (beef and Nellore) were provided access to grains or concentrates, they spent less time grazing compared to calves provided no concentrated feed [77,78]. However, there is evidence suggesting that pasture is a valued resource for calves. For instance, despite considerable differences in individual grain intake, dairy calves raised on pasture consumed less grain pre-weaning than calves raised in a barn (pasture: 19.7 kg, barn: 29.3 kg), suggesting they forwent grain for grass [98]. More recent work has also found that providing fresh cut ryegrass to individually housed calves (mixed breeds) indoors reduced their concentrate intake [99]. Though pasture management and supplementation has continued to be studied in beef calves, the focus has been on calf production measures with little attention to calf behaviour [100,101].

An opportunity to graze may allow calves more agency over their dietary choices. Some authors have argued that offering diverse diets allows each animal to play an active role in fine tuning their diet, perhaps reducing the risk of certain maladies and avoiding satiety to single food [102]. Access to a variety of feeds and making choices about which food to eat may allow an animal to regulate individual dietary needs [103,104,105]. For example, dairy calves housed indoors with free choice to different feeds showed individual variation in their dietary choices that led to few non-nutritive oral behaviours [105], while still meeting their nutritional needs [106]. The next step may be to describe the agency a calf may exercise over what he or she eats when provided access to pasture.

In the US, most (70%) pre-weaned heifer calves are housed in individual hutches; the rest are housed in tie stalls (5%), freestalls (3%), open/dry lots (3%), or as a group in a barn (15%) [29]. In Canada, individual hutches (21%), individual pens (40%), group housing (35%), and tethering (2%) are used to house pre-weaned heifer calves [69]. Dairy calf housing is very different from the expansive and variable environment described for calves in natural systems. The United States Department of Agriculture (USDA [29]) reported that 43.8% of pre-weaned heifer calves are raised outside, mostly in hutches; but only 1.6% are given pasture access. In southern Brazil, 13% of dairy calves are provided access to pasture [28]; in Australia, 42.4% of calves have outdoor access [30]; and in Uruguay, 97.9% of calves are provided outdoor access usually with shade and shelter [107]. Despite some countries providing outdoor access to their calves, there is little information about the preferences, development, and opportunities calves may have when given the choice to go outside.

#### 2.2.9. Rumination

Though absent in newborn calves, development of rumination allows calves to use solid feed and transition from a milk diet [108]. One study reported that 4 h after access to pasture, dairy calves 1 to 2 wk old (without their mothers) began ruminating [98]. At 2–3 wk of age, beef calves without their mothers ruminated for approximately 1 h/d after only 3 d on pasture [77], and rumination in zebu calves with their mothers was not reliably recorded until they were 18 d old [45]. These slight discrepancies could be due to differences in definition, or the difficulty in reliably recording rumination without cameras or other computer aids. However, all studies agreed that calves spent more time ruminating with age due to increased intake of solid food. Zebu calves at 2 mo of age spend about 6 h/d ruminating, and by 6 mo, calves will be ruminating about 9 h/d when on pasture [45]. Rumination may also be affected by the type of feed available to the calf. Much of the research on dairy calf rumination focuses on calf growth and rumen physiology when young calves are provided hay [108,109,110]. One study compared behaviour and rumen development when group-housed veal calves were provided unlimited hay or other roughages (i.e., straw, maize silage, or maize cob silage); calves fed hay spent the most time chewing and ruminating and the least amount of time orally manipulating their environment [111]. However, pre-weaned calf rumination on pasture has received less attention. One study compared a concentrate diet and straw to concentrates and fresh cut grass, and found that calves fed grass spent more time ruminating, likely because this palatable forage stimulated rumen development [99].

### 2.3. Weaning

Weaning age has been studied in a few different natural settings. Free-ranging reindeer calves appear to be completely weaned from milk at 6–7 mo of age [24], water buffalos wean at approximately 1 year of age [36], yearling beef calves are refused suckling after the new calf is born [112], and Zebu and Buran female calves wean at 9 mo of age, and males wean at 11 mo of age [48,49]. Calves appear to retain an affinity towards their mothers and siblings after weaning. When beef calves were reared with continued maternal contact, yearling calves had more non-agonistic encounters with their newborn sibling than with non-related newborn calves [112]. Similarly, buffalo calves remain in the herd for at least 2 years, and the yearling calf is frequently in close contact with the younger sibling [36]. When calves are weaned from milk, they appear to strengthen bonds with other social contacts, perhaps to compensate for the loss of maternal contact [113].

Different from natural settings, where weaning from the mother is a gradual process (culminating when the calf is 7 to 14 mo old, and involves a series of events such as the gradual decrease in milk, a steady increase in solid food consumption, and the mother beginning to reject some of the calf’s suckling attempts; reviewed by Enríquez et al. [114]), dairy calves are usually weaned abruptly, with milk feeding stopped by 9 wk of age [29]. Abrupt weaning is defined as immediate milk removal, such that calves are fed their full milk allowance (e.g., 9 L/d), until the day of weaning, where milk is immediately removed (0 L/d) [115,116,117], or calves remain in full contact with their mothers until the day of weaning, where calves are immediately removed from their mother with no contact [118]. Gradual weaning is often described as some form of a step-down procedure where calves are provided their full milk allowance until 3–6 wk of age, at which point the milk is gradually reduced over 5 d to half the volume (e.g., 8 L/d to 4 L/d over 5 d); calves are fed this reduced volume until weaning where milk is once again reduced over 5 d (to 0 L/d) [119,120,121]. Other studies have described gradual weaning as a gradual decrease in milk allowance over 4–22 d [115,117], or a gradual decrease in maternal contact with nose-flaps or fence-line weaning [118,122]. Abrupt weaning may cause challenges such as depressed growth and increased distress behaviours such as walking and vocalizations, compared to calves that are gradually weaned (reviewed by Enríquez et al. [114], and Khan et al. [91]). Experimental work suggests that the behavioural response to weaning can be to both loss of milk and loss of access to other aspects of the milk feeding routine; for example, when artificially reared, nipple-fed calves were weaned from milk by replacing the milk with warm water from a teat, calves were less active and vocal compared to control calves that no longer had access to milk or a teat [123]. Similarly, compared to calves who were abruptly weaned from their mothers, beef calves weaned in 2 steps (first calves were fitted with nose flaps that prevented suckling while still allowing calves access to the cow, then calves were separated from the cow) performed fewer vocalisations, walked less, and spent more time lying and eating [122].

In addition to management, there may also be individual differences in feeding behaviour [124] that can affect responses to weaning. For instance, individual Holstein calves raised indoors vary considerably in age (from 23 to 82 d) when they start eating solid food (200 g/d) [125], and when they wean based on solid food intake [125,126,127]. These individual differences in feeding behaviour may be due to personality traits such as exploration and fearfulness [124]. However, given the complex social behaviours that have been described during weaning in natural settings, future work should also explore the role of sociability (a trait associated with feeding behaviour in adults [128]) in individual feeding behaviour and weaning.

During weaning, many dairy calves in North America are alone [29]. In more natural systems, groups are usually made up of mixed ages; a social environment may be especially important at weaning. For example, dairy calves living with an older weaned companion visited the starter feeder more frequently and gained more weight than calves in a single age group [129]. The older social companion may have facilitated social learning [55] and also provided social support (i.e., calves experiencing stressful situations may have derived support from other calves [130]).

## 3. Implications for Management

As reviewed, calves in natural systems (i.e., on pasture in herds with their mothers) have access to a complex social group and varying physical features that affect their behavioural development. In contrast, most dairy calves are raised individually without varying physical features. Though dairy farms can be limited by space and season, there may be practical ways to support calf behaviour. This review highlights the importance of the mother and other adults on the development of the calf, particularly feeding and social development. Research on ways to improve cow and calf contact has focused on the birthing period [14,15,42], and more work on longer-term dairy cow–calf contact systems is needed [35,131]. Though a complex social group is important for calf development, we will focus on recommendations for calves raised without adults.

To better meet the normal behavioural development of the pre-weaned dairy calf, some management considerations that foster a calf’s key natural behaviours may be implemented to improve their welfare. Calves spend most of their time lying, thus all calves should have access to clean, dry areas for resting [132,133]. Allogrooming appears to be an important behaviour for calves [33], though calf–calf grooming is infrequent [59]. Calves value brushes [61], and farms may be able to encourage grooming by offering a brush to calves, even using simple designs [61]. Other calf activities such as walking and play may be supported by access to a larger space [134]. Some dairy farms may not have the infrastructure to manage calves in large pens; for these farms, one option is to offer calves access to “play pens” in the evenings to accommodate diurnal behavioural patterns and promote play and walking behaviours.

As calves are herd animals, providing social partners soon after birth is required for their behavioural development [72,76,135] during their first two months of age. To our knowledge, no study has explored the long-term effects of early social housing on the behavioural development, cognition, and coping abilities of adult cattle. Only one study suggests that early social housing has no advantage or disadvantage for adult performance, longevity, or activity when an adult was regrouped [136]. Future research may consider exploring the long-term effects of early social housing for calves. Given the numerous welfare benefits of early social housing for the young calf (see reviews by Costa et al. [137] and Cantor et al. [6]), we recommend that farms house pre-weaned calves together, and that future research consider the effects of housing dairy calves in mixed-age herds with their mothers. Housing calves in pairs can be carried out using existing farm infrastructure (e.g., hutches [138,139]). The health of calves in groups less than 10 can be managed [140], and automatic milk feeding systems can promote natural sucking of group housed calves and allow them to receive higher cumulative volumes of milk [70]. One concern raised for social housing calves is cross-sucking [138]; however, this behaviour is more often observed in calves fed milk in restricted quantities [87] and by bucket [85]. Thus, calves should be fed high milk volumes (ad libitum encouraged) from a teat (i.e., their mother, teat bottle, or automatic milk feeder) to support natural sucking [80,84,85].

Weaning is a challenge for calves, especially for those fed high milk allowances, so calves can benefit from gradual weaning, where milk is reduced over time to mimic the natural transition from milk to solid feed [91]. Gradual weaning can be carried out by programming automatic milk feeders, or gradually decreasing the quantity of milk offered to encourage solid feed intake [91,120]. Automatic milk feeders can also be utilized to consider each individual calf’s feeding behaviour, such that calves can be gradually weaned based on reaching intake targets for solid feed [126,127]. Additionally, though dairy calves are often weaned at 9 wk of age [29], farms considering more natural management (weaning at 7–14 mo [24,36,49]) may offer a prolonged milk feeding program. In summary, dairy farms should consider gradual weaning programs tailored to the needs of individual calves.

Finally, to promote a more natural time budget, and aid in the transition from milk to solid feed, dairy systems should consider offering pre-weaned calves pasture access. Calves begin grazing at a young age [45,77], and access to grass may be beneficial for rumen development [99]. Some pasture systems such as those in Australia and New Zealand [30,141] manage pre-weaned calves with pasture access, suggesting that these methods could also be adapted elsewhere.

## 4. Conclusions

Calves reared with their mothers and herd-mates in more naturalistic systems show a breadth of behaviours. These calves slowly transition from their mother’s milk to solid feeding by increasing time spent grazing and ruminating while decreasing time spent lying and playing. In contrast, on many dairy farms, the calf is removed from the mother at birth, housed individually, fed limited amounts of milk and weaned abruptly at a young age. Approaches to accommodate natural living include the use of nipple feeding with higher milk allowances, social housing, gradual weaning, and access to grazing opportunities.

## Data Availability

Not applicable.
